# Challenging age limits: favourable mid-term outcomes of periacetabular osteotomy in patients over 30 years of age with borderline hip dysplasia

**DOI:** 10.1093/jhps/hnaf061

**Published:** 2025-10-15

**Authors:** Maximilian Fischer, Andreas Nitsch, Alexander Möller, Andre Hofer, Georgi I Wassilew

**Affiliations:** Center for Orthopaedics, Trauma Surgery and Rehabilitation Medicine, University Medicine Greifswald, Ferdinand-Sauerbruch Straße, Greifswald 17475, Germany; Center for Orthopaedics, Trauma Surgery and Rehabilitation Medicine, University Medicine Greifswald, Ferdinand-Sauerbruch Straße, Greifswald 17475, Germany; Center for Orthopaedics, Trauma Surgery and Rehabilitation Medicine, University Medicine Greifswald, Ferdinand-Sauerbruch Straße, Greifswald 17475, Germany; Center for Orthopaedics, Trauma Surgery and Rehabilitation Medicine, University Medicine Greifswald, Ferdinand-Sauerbruch Straße, Greifswald 17475, Germany; Center for Orthopaedics, Trauma Surgery and Rehabilitation Medicine, University Medicine Greifswald, Ferdinand-Sauerbruch Straße, Greifswald 17475, Germany

**Keywords:** developmental dysplasia of the hip, borderline hip dysplasia, periacetabular osteotomy, patient-reported outcomes, age

## Abstract

The treatment of borderline hip dysplasia (BHD) remains controversial, with a paucity of literature on whether periacetabular osteotomy (PAO) is a viable option even in middle-aged patients over 30 years of age. This study aimed to analyse the functional outcome and activity level in relation to the patient’s age after PAO for BHD. Patients over 30 years of age (G30—age 30–40; G40 age > 40) undergoing PAO for BHD were enrolled in this single-centre study with a minimum follow-up of 2 years. The patient-reported outcomes (PRO) were assessed using the numeric rating scale (NRS) for pain, the modified Harris-Hip score (mHHS), and International Hip Outcome Tool-12 (iHot-12) for joint functionality, and the University of California Los Angeles activity score (UCLA) for sports participation. Forty-eight patients (48 hips) were included. There was a significant postoperative pain relief in both groups (G30 pre-post 7.2 - 2.7, *P* < .001; G40 pre-post 7.3 - 2.5, *P* < .001). Joint functionality improved postoperatively with significant higher scores for mHHS (G30 pre-post 48.0 - 80.0, *P* < .001; G40 pre-post 44.9 - 84.4, *P* < .001) and iHot-12 (G30 pre-post 40.1 - 68.8, *P* < .001; G40 pre-post 41.5 - 64.9, *P* = .004) for patients older than 30 and 40 years. Sports participation only improved significantly after PAO in patients older than 40 years (pre-post 5.4 - 6.9, *P* = .019). Overall, PAO is a viable option to treat BHD in middle-aged patients, while improving pain and joint function. Even patients over 40 years of age can benefit in terms of activity and joint function.

## INTRODUCTION

Hip preservation by periacetabular osteotomy (PAO) has become an increasingly successful and widely adopted treatment for both severe and borderline hip dysplasia (BHD) [1–3]. In this respect, several individual patient factors affecting the postoperative results after PAO have been identified in patients with severe hip dysplasia, such as the patient’s sex or advanced cartilage damage [4–6].

Conflicting results exist regarding the patient’s age as relevant factor for potentially inferior PAO outcomes, and this has already been studied in cohorts with severe hip dysplasia. For instance, Leopold *et al.* reported improved patient-reported outcomes (PRO) even in middle-aged patients, whereas long-term data by Willey *et al.* emphasized that an increased patient’s age represents a relevant factor for poor PRO or conversion to total hip arthroplasty [7, 8]. As treatment recommendations in BHD ranging from hip arthroscopy to the more invasive PAO, this topic is of substantial importance in preoperative patient guidance [9, 10]. A recent meta-analysis indicated comparable outcomes for PAO and hip arthroscopy in BHD but indicated a potential advantage of PAO, as it provides definitive correction of the anatomical deformity [11]. However, the role of the patient’s age affecting PRO in patients undergoing PAO for BHD remains poorly studied.

In general, studies on PRO in BHD cohorts are mainly based on small cohorts reporting good results, even without stratification on patient individual factors affecting the postoperative results [12, 13]. Nepple *et al.* reported improved PRO after PAO in their study on 186 BHD hips and identified previous hip arthroscopy as a relevant risk for inferior PAO outcomes [14]. In this respect, further stratification of patients by age may help determine whether the invasive PAO is a viable therapeutic option for older patients with BHD. In addition, interpreting the clinical relevance of PRO scores can substantially improve preoperative patient guidance on postoperative outcome expectations. In this context, minimum clinically important difference (MCID), substantial clinical benefit (SCB), and patient-acceptable symptom state (PASS) have become prominent metrics for evaluating the clinical significance of PRO [15].

As a result, this study aimed to comprehensively evaluate PRO in a cohort of 48 middle-aged patients older than 30 years treated by PAO for BHD, with a minimum follow-up of 2 years. It is hypothesized that even middle-aged patients with BHD can be successfully treated with PAO, achieving substantial improvement in joint functionality and activity level.

## MATERIALS AND METHODS

### Study design

Forty-eight patients (48 hips) undergoing PAO for symptomatic BHD with an age over 30 years were identified from the institutional PAO registry and included for final analysis in this single-centre study. The patients underwent PAO due to symptomatic BHD [defined by a lateral centre-edge angle (LCEA) 18°–25°] without or only minimal signs of osteoarthritis (Tönnis grade 0–1) between May 2019 and January 2023 (Fig. 1). The LCEA cut-off values were determined based on a large cohort study of patients with BHD [14].

**Figure 1:**
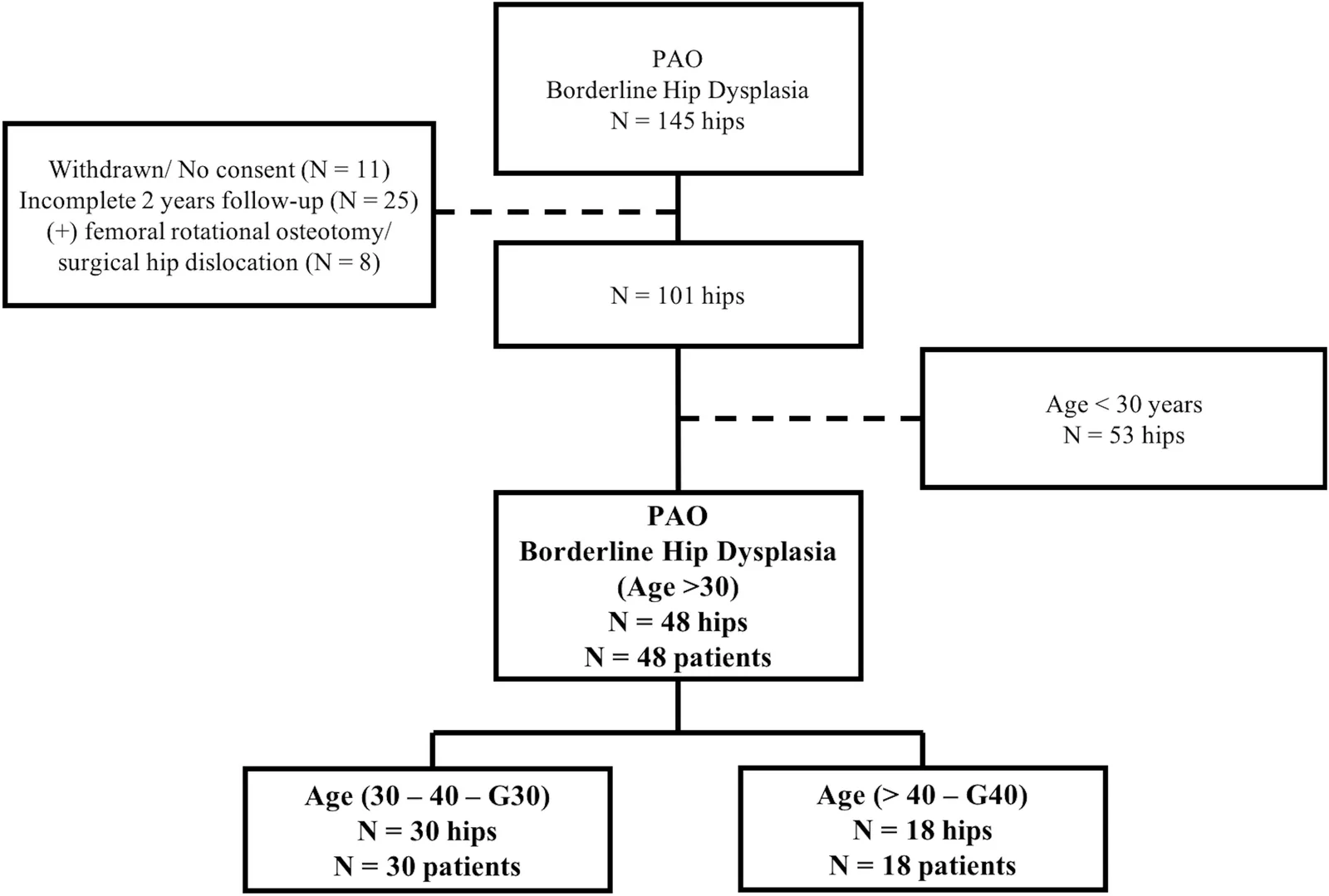
Flow-chart of included patients.

All included patients completed the PRO assessment and had a minimum follow-up of 2 years. The mean follow-up was 4.2 years.

The patients were divided in accordance to their age at the time of surgery into two groups G30 (age 30–40 years, *n* = 30) and G40 (age > 40 years, 40–49.5 years, *n* = 18) to evaluate PRO.

### Radiographic assessment and surgical technique

Preoperative and postoperative anteroposterior pelvic radiographs were reviewed independently to analyse Tönnis osteoarthritis grade, LCEA, Tönnis angle (TA), as well as anterior- and posterior wall index (AWI/PWI) (see Table 1). A modified, minimally invasive Bernese PAO technique was performed in every patient. All procedures were done by one fellowship-trained, high-volume surgeon (GIW). Physiotherapeutic training and mobilization of the operated hip joint started directly on the first postoperative day with partial weight-bearing until six weeks postoperative.

**Table 1:** Patient demographics and acetabular morphology.

	Total (*n* = 48)	G30 (*n* = 30)	G40 (*n* = 18)	*P*
Age ± SD	37.65 (± 5.46)	33.91 (± 2.31)	43.88 (± 2.89)	<.001
BMI ± SD	25.59 (± 4.12)	25.90 (± 4.30)	25.08 (± 3.73)	.748
*n* (% female)	42 (87.5)	24 (80)	18 (100)	.115
LCEA (° ± SD)	20.41 (± 2.23)	20.56 (± 2.20)	20.16 (± 2.24)	.456
TA (° ± SD)	8.53 (± 4.36)	9.10 (± 3.19)	7.56 (± 5.69)	.514
AWI (± SD)	0.43 (± 0.11)	0.44 (± 0.12)	0.43 (± 0.09)	.879
PWI (± SD)	0.91 (± 0.16)	0.92 (± 0.17)	0.89 (± 0.15)	.616
Post LCEA (° ± SD)	31.32 (± 3.94)	31.63 (± 4.09)^a^	30.85 (± 3.62)^a^	.644
Post TA (° ± SD)	−0.24 (± 4.31)	0.13 (± 3.91)^a^	−0.85 (± 4.85)^a^	.739

SD, standard deviation. LCEA, lateral centre-edge angle. AWI/PWI, anterior/posterior wall index. ^a^significant intragroup difference compared to preoperative, *P* < .05

### PRO assessment and statistical analysis

PRO were assessed during routine follow-up examination. The patients’ pain level was assessed by a numeric rating scale (NRS) with 0 (best) and 10 (worst) scores. The modified Harris-Hip score (mHHS) and the International Hip Outcome Tool-12 (iHot-12) were used to quantify hip function [16, 17]. The University of California Los Angeles (UCLA) activity score determined patient-reported sports participation [18].

For clinical interpretation of the reported PRO, the mHHS was used to determine the proportion of patients achieving the MCID (threshold 18), SCB (threshold 23), and PASS (threshold 71), based on thresholds previously defined for PAO cohorts [15].

Continuous variables and PRO are presented as mean and standard deviation, while categorical variables are presented as absolute numbers and percentages.

Statistical analysis and visualization were performed using SPSS (version 29, IBM, Armonk, NY, USA) and GraphPadPrism (version 10.3, GraphPad Software, Boston, MA, USA). The preoperative and postoperative intragroup PRO analyses were conducted using a paired t-test. Intergroup differences were tested by Mann–Whitney *U* test for continuous variables and chi-square with Yates ‘continuity correction test for categorial variables. A *P*-value < .05 was considered statistically significant.

### Ethical considerations

All included patients gave written informed consent prior to inclusion. Ethics approval (BB099/20a) was obtained from the local independent ethics committee of the University Medicine Greifswald according to the World Medical Association Declaration of Helsinki.

## RESULTS

Femoral head coverage improved after PAO with a significant increase in LCEA (G30/40, *P* < .001), as well as an accompanied decrease in TA (G30/40, *P* < .001) in both age groups (see Table 1).

At the final follow-up, G30 (pre-post 7.2 - 2.7, *P* < .001) and G40 (pre-post 7.3 - 2.5, *P* < .001) reported significant pain relief after PAO (see Fig. 2A). Furthermore, G30 showed a significant improvement in mHHS (pre-post 48.0 - 80.0, *P* < .001) and iHot-12 (pre-post 40.1 - 68.8, *P* < .001) (see Fig. 2B/C). In line with this, there was even a significant improvement in joint functionality in patients older than 40 years of age (mHHS pre-post 44.9 - 84.4, *P* < .001; iHot-12 pre-post 41.5 - 64.9, *P* = .004) (see Fig. 2B/C). A significant postoperative improvement in UCLA score was only found for the G40 group (pre-post 5.4 - 6.9, *P* = .019) (see Fig. 2D).

**Figure 2:**
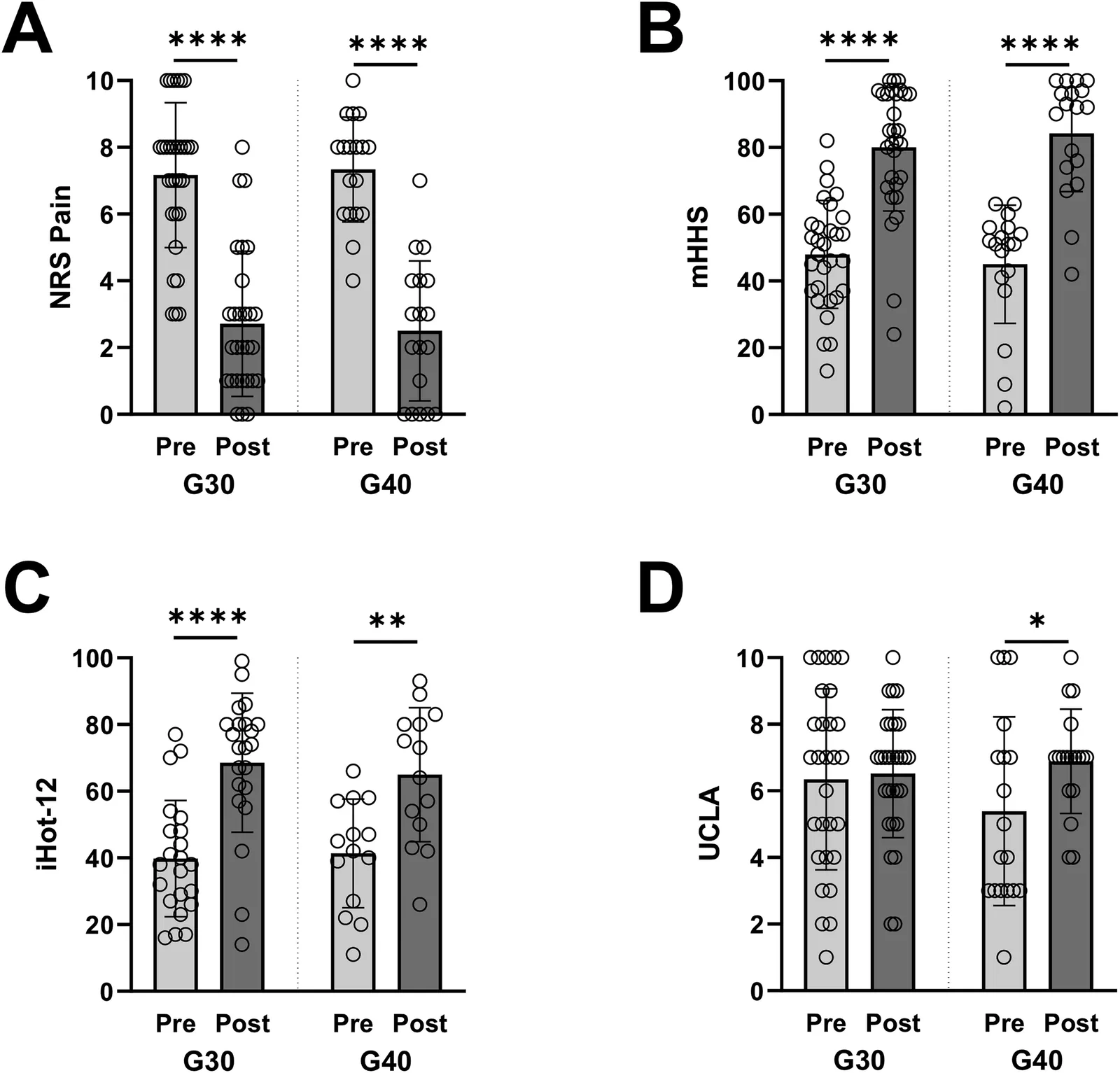
PRO before and after PAO.

83% of patients older than 40 years of age achieved the MCID, while 77% in the group G30 achieved the postoperative MCID at the latest follow-up (see Table 2). Additionally, 73% of G30 achieved SCB and PASS after PAO. Patients aged older than 40 years achieved SCB and PASS in 77% of cases (see Table 2).

**Table 2:** Interpretation of postoperative PRO.

**mHHS**	Cut-off value	G30% achieved (x/30)	G40% achieved (x/18)
MCID	18	77 (23/30)	83 (15/18)
SCB	23	73 (22/30)	77 (14/18)
PASS	71	73 (22/30)	77 (14/18)

Postoperative achievement of MCID, SCB, and PASS using the mHHS. MCID, minimum clinically important difference. SCB. substantial clinical benefit. PASS, patient-acceptable symptom state.

There were no major complications after PAO in both groups. The most frequent complication was patient-reported numbness at the lateral thigh due to lateral femoral cutaneous nerve lesion (66% versus 50%, *P* = .40). Furthermore, every group showed one case of inferior pubic ramus stress fracture (3% versus 6%, *P* = .72) and one case of pubic non-union (3% versus 6%, *P* = .72), all without need for revision surgery (Table 3).

**Table 3:** Postoperative complications.

Complications	G30 % events (x/30)	G40 % events (x/18)	*P*
Deep vein thrombosis	0 (0/30)	0 (0/18)	1
Pulmonary embolism	0 (0/30)	0 (0/18)	1
Wound healing disorders	6.7 (2/30)	0 (0/18)	.70
Nerve injuries Motor nerve Sensory nerve	0 (0/30) 66 (20/30)	0 (0/18) 50 (9/18)	1 .40
Stress fracture	3 (1/30)	6 (1/18)	.72
Non- union	3 (1/30)	6 (1/18)	.72

## DISCUSSION

This cohort study on 48 hips in 48 middle-aged patients older than 30 years undergoing PAO for BHD aimed to analyse the functional outcome and activity level in relation to the patients’ age at mid-term follow-up. The main findings of this study were (I) PAO improves pain and joint functionality in middle-aged patients with BHD; (II) more than three out of four patients older than 40 years’ experience a substantial benefit when BHD is treated by PAO, and (III) PAO in middle-aged patients is a safe surgical procedure with low complication rates.

Insufficient femoral head coverage in hip dysplasia is a well-described risk factor for increased joint degeneration over time, with the relative risk increases as the LCEA decreases. Therefore, early hip preservation intervention is recommended to positively influence the hip joint survival [19]. Data from a meta-analysis reported joint survival rates of over 80% at 15-year follow-up after PAO in patients under 40 years of age with hip dysplasia [20]. Hence, PAO in BHD may positively influence the natural hip joint even in older patient cohorts.

The data of the current study found that patients older than 30 years with symptomatic BHD achieve a substantial improvement in patient-reported joint functionality and activity level after PAO. Furthermore, there was no increase in inferior outcomes with increasing patient age. This is in line with data by Leopold *et al.* reporting PRO after PAO for severe hip dysplasia compared to the current BHD cohort. In their cohort study on 120 hips, the authors found a constant postoperative improvement in PRO comparing young and middle-aged patients [7]. Thus, the data of the current study extend the knowledge on PRO in middle-aged patients and found that PAO is a viable treatment option even in BHD.

Nevertheless, there are several reports on higher rates of PAO failure with increasing patients age. For instance, Larsen *et al.* reported in their large-scale and long-term follow-up study a higher rate of treatment failure with increasing patients age. In contrast to the current study, the primary endpoint was set on the conversion to total hip arthroplasty, while the current study focused on PRO [21]. However, even when only reporting on a small cohort of middle-aged patients with BHD in the current study at mid-term follow-up, there were no conversions to total hip arthroplasty during the study period.

Surprisingly, sports participation was only significant improved after PAO in patients older than 40 years. One potential reason for this finding could be the different proportion of female patients in the G30 and the G40 group. The patients’ sex was found to represent a relevant factor affecting the postoperative outcomes after PAO [4]. In this respect, data from the ANCHOR study group of 359 hips demonstrated that females experience a higher improvement in sports participation compared to their male counterparts after PAO [22]. Thus, interpreting the current study results on sports participation in patients older than 40 years may be biassed due to a higher rate of females in this study group.

Furthermore, the current study aimed to further clinically interpret the reported PRO, and it was found that nearly 80% of middle-aged patients achieved a meaningful change in their health when BHD was treated by PAO. Additionally, three out of four patients experience a substantial benefit after the operation. These results are higher than reported by Trotzky *et al.,* even when their results were reported with a mean follow-up of 1 year, and the current study results reporting mid-term outcomes [15]. Thus, rehabilitation after PAO may extend beyond 1 year postoperatively, and patients can continue to experience functional improvement even longer after the initial postoperative phase. Compared to studies on BHD cohorts at mid-term follow-up treated by hip arthroscopy, slightly higher scores for achieving MCID and PASS were reported by Beck *et al.* after hip arthroscopy [23]. It has to be mentioned that the cut-off values for MCID and PASS were set on other levels, and this could potentially explain the different results of the current study.

Hence, it becomes clear that studies directly comparing hip arthroscopy and PAO in BHD cohorts would be the next step to improve the evidence of treatment recommendations for BHD.

In this respect, the risk of complications and invasiveness of both procedures must be considered in preoperative guidance of patients with BHD. Even when there are technical improvements with muscle-sparing, minimally invasive approaches, there is still a risk of intraoperative complications after PAO [24, 25]. A systematic review on a large cohort undergoing PAO reported an overall complication rate of 7% even with severe complications, including extended blood loss or nerve injuries [26, 27]. Besides systemic complications, bone-related complications regarding stress fractures and non-union were reported to be associated with an increasing patient’s age [5]. In contrast, the current study found no severe complications and low rates of postoperative stress fractures or non-union. Thus, PAO shows high safety even in middle-aged patients. In this cohort, lateral femoral cutaneous nerve lesions were the most frequent postoperative complication, with an incidence of 50% and 66%, respectively. This is in line with prospectively collected data by Doi *et al.* on 42 PAO patients. However, despite the high incidence of femoral cutaneous nerve lesions, the authors reported that postoperative PRO were not negatively influenced in their study. This is consistent with the findings of the current study in BHD, showing a significant improvement in joint functionality after PAO [28].

Nevertheless, meticulous preoperative guidance and a critical evaluation of the expected outcome based on individual patient factors are necessary as shown in the current study. Consequently, studies on patient individual factors affecting the PRO must be extended to subgroups of hip dysplasia, like BHD, to improve the PAO patient care. The current study contributes to this knowledge on postoperative outcome expectations and reported successful mid-term results in middle-aged patients undergoing PAO for BHD.

However, several limitations of this study must be considered and discussed. First, the current study focused on a selected patient cohort with a limited number of cases included. In this respect, studying the patients’ age affecting the postoperative results in patients with other indications for PAO, for instance, acetabular retroversion, could improve the generalizability of the study results. Especially, the small number of patients older than 40 years limits the available evidence in this age group. Nevertheless, the combination of BHD and higher patient age represents a rare condition, and the results may serve as a starting point for future studies on PAO outcomes in this age group. In this respect, the favourable mid-term outcomes shown in this study have to be verified in ongoing long-term evaluations on large patient BHD cohorts. Next, the current study included only patients receiving PAO to treat BHD, and there could be an increased risk for selection and treatment bias caused by a single surgeon performing all treatments in this study. Therefore, prospective multicentre studies comparing hip arthroscopy and PAO could improve the evidence for treatment recommendations in middle-aged BHD patients.

## CONCLUSION

Overall, this cohort study indicates that higher patients age alone is not a contraindication for PAO in BHD patients, while leading to improved mid-term outcomes in joint functionality and activity level. Over 70% of patients older than 40 years experience substantial individual improvements when BHD is treated with PAO. Furthermore, even in older patients, PAO can be performed safely with low complication rates.

## Data Availability

The datasets generated and analysed in the current study are not publicly available due to data protection regulations. Access to data is limited to the researchers who have obtained permission for data processing. Further inquiries can be made to the corresponding author.
